# Data on COA-Cl administration to the APP/PS2 double-transgenic mouse model of Alzheimer׳s disease: Improved hippocampus-dependent learning and unchanged spontaneous physical activity

**DOI:** 10.1016/j.dib.2018.09.044

**Published:** 2018-09-19

**Authors:** Yasushi Kishimoto, Ikuko Tsukamoto, Atsuko Nishigawa, Akiko Nishimoto, Yutaka Kirino, Yoshihisa Kato, Ryoji Konishi, Tokumi Maruyama, Norikazu Sakakibara

**Affiliations:** a Kagawa School of Pharmaceutical Sciences, Tokushima Bunri University, Japan; b Department of Pharmaco-Bio-Informatics, Faculty of Medicine, Kagawa University, Japan

**Keywords:** AD, Alzheimer׳s disease, APP, amyloid precursor protein, MWM, Morris water maze, PS2, presenilin 2, Alzheimer׳s disease, Angiogenesis, Novel object cognition test, Spatial memory, Spontaneous physical activity, Non-spatial memory

## Abstract

We herein present behavioral data regarding whether COA-Cl, a novel adenosine-like nucleic acid analog that promotes angiogenesis and features neuroprotective roles, improves cognitive and behavioral deficits in a murine model for Alzheimer׳s disease (AD). COA-Cl induced significant spatial memory improvement in the amyloid precursor protein/presenilin 2 double-transgenic mouse model of AD (PS2Tg2576 mice). Correspondingly, non-spatial novel object cognition test performance also significantly improved in COA-Cl-treated PS2Tg2576 mice; however, these mice demonstrated no significant changes in physical activity or motor performance. COA-Cl did not change the spontaneous activities and cognitive ability in the wild-type mice.

**Specifications table**

**Table:** 

Subject area	*Neuroscience*
More specific subject area	*Alzheimer׳s disease, behavioral neuroscience*
Type of data	*Graph, figure*
How data was acquired	*Behavioral phenotyping (HomeCageScan, TopScan; CleverSys Inc., Reston, VA,) with Morris Water Maze, Novel object recognition test, and Spontaneous physical activity.*
Data format	*Analyzed*
Experimental factors	*5- to 6-month-old APP/PS2 double-transgenic (PS2Tg2576) mice, littermate wild-type mice, COA-Cl.*
Experimental features	*Changes in behavior and memory of PS2Tg2576 and control wild-type mice following COA-Cl or PBS treatment were characterized.*
Data source location	*Sanuki-shi, Kagawa, JAPAN*
Data accessibility	*Data within this article*
Related research article	*Involvement of S1P1 receptor pathway in angiogenic effects of a novel adenosine-like nucleic acid analog COA-Cl in cultured human vascular endothelial cells,* Pharmacology Research & Perspectives*,***2:** e00068.

**Value of the data**

• These data demonstrate the effects of a novel adenosine-like nucleic acid analog COA-Cl on behavior and cognition of a murine model of Alzheimer׳s disease (AD).
• These data are useful for investigators researching the pharmacological and physiological effects of COA-Cl.
• These data provide grounds for further study on the potential use of COA-Cl to treat neurodegenerative diseases.
• These data provide additional evidence for angiogenesis as a future therapeutic strategy to treat AD.

## 1 Data

**Fig. 1:**
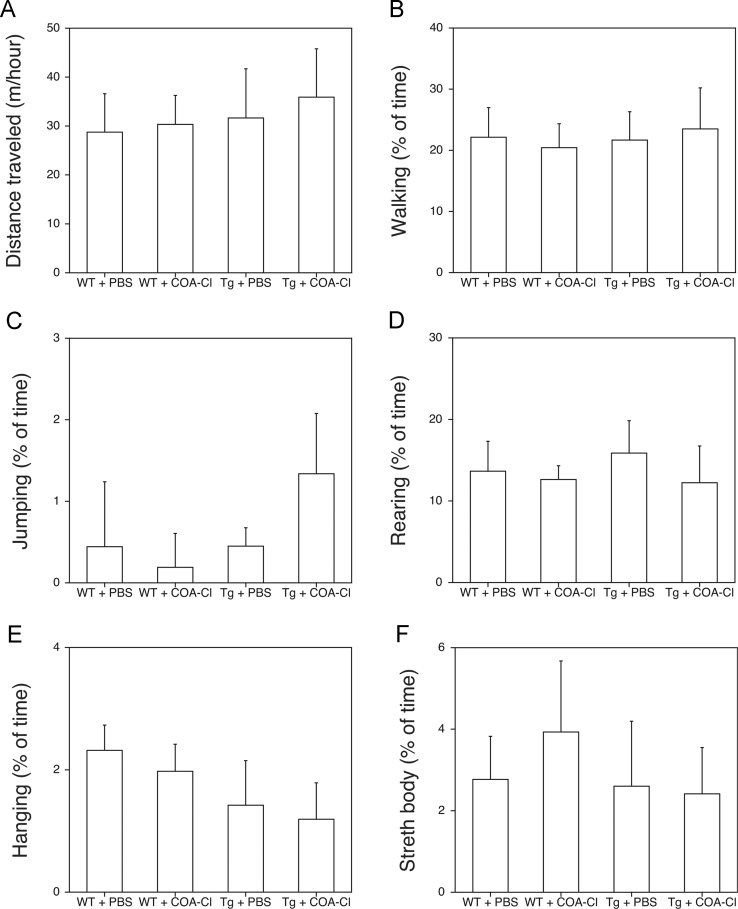
Data showing the six spontaneous physical activities performed by the wild-type and PS2Tg2576 mice following COA-Cl or PBS treatment in their home cages: (A) distance traveled, (B) walking, (C) jumping, (D) rearing, (E) hanging, and (F) body stretching. Data points represent the mean ± SEM. The number of mice in each group was 8.

**Fig. 2:**
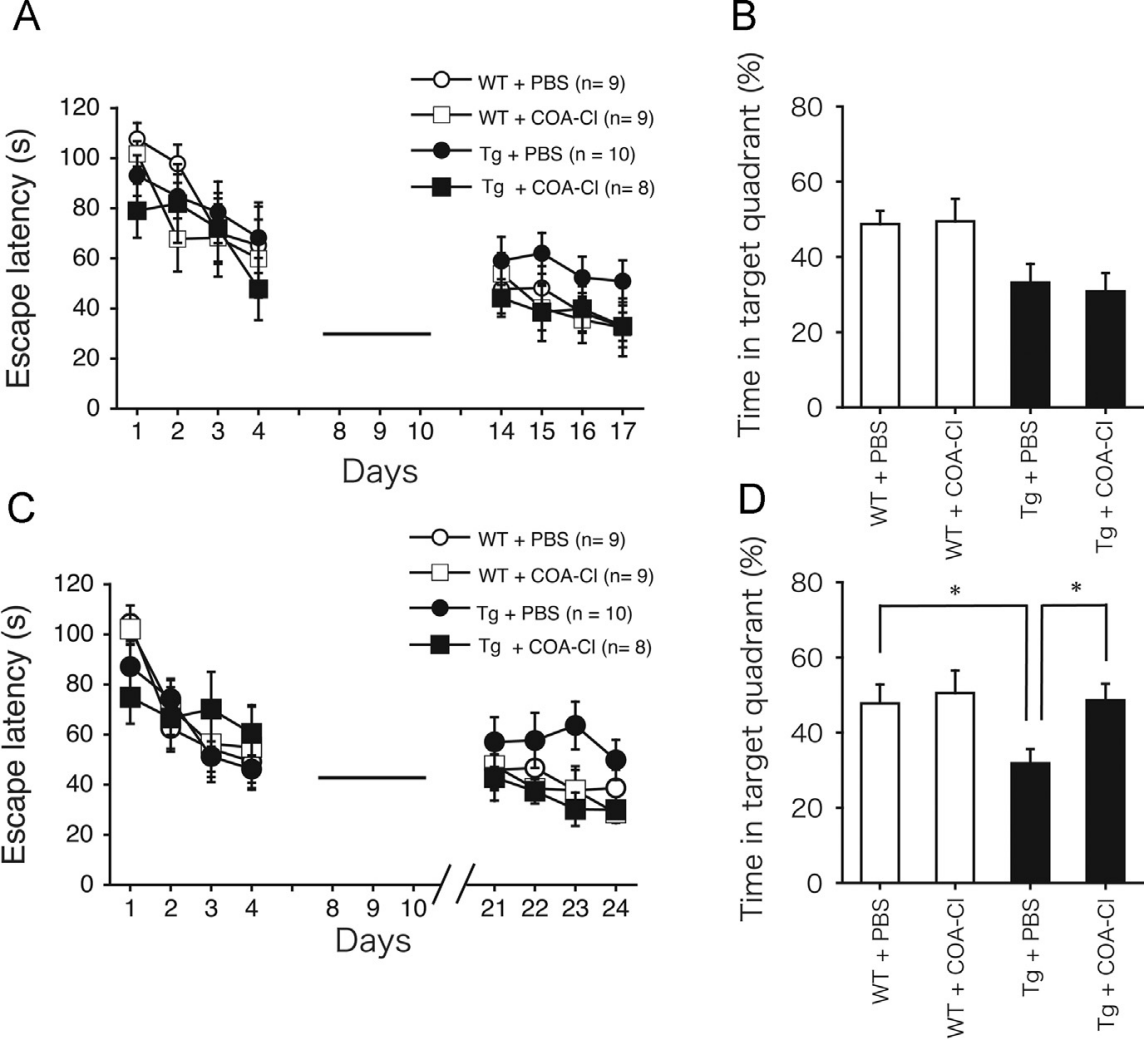
Data showing improvement of spatial-memory deficits in PS2Tg2576 mice after COA-Cl treatment. (A and B) MWM test 1. (A) The escape latencies in the MWM during the training and behavioral testing phases (days 1–4 and 14–17, respectively) were evaluated in PBS-treated WT mice (open circle, *n* = 9), COA-Cl-treated WT mice (open square, *n* = 9), PBS-treated PS2Tg2576 mice (closed circle, *n* = 10), and COA-Cl-treated PS2Tg2576 mice (closed square, *n* = 8). The mean values of the escape latencies during behavioral tests were not significantly different between these four groups. ANOVA revealed no significant interaction effects between sessions and groups [*P* = 0.98; *F*(9, 96) = 0.28] and no significant group effect [*P* = 0.23; *F*(3, 32) = 1.49]. (B) Probe trial data. The percentage of the total time spent in the target quadrant was significantly different between the four groups [*P* = 0.027; *F*(3, 32) = 3.48]; however, there was no significant difference between PBS-treated PS2Tg2576 mice and COA-Cl-treated PS2Tg2576 mice. (C and D) MWM test 2. (C) The escape latencies in the MWM during the training and behavioral testing phases (days 1–4 and 21–24, respectively) were evaluated in PBS-treated WT mice (open circle, *n* = 9), COA-Cl-treated WT mice (open square, *n* = 9), PBS-treated PS2Tg2576 mice (closed circle, *n* = 10), and COA-Cl-treated PS2Tg2576 mice (closed square, *n* = 8). The escape latencies of were not significantly between these for groups. ANOVA revealed no significant interaction effects between sessions and groups [*P* = 0.86; *F*(9, 96) = 0.52], and no significant group effect [*P* = 0.042; *F*(3, 32) = 0.091]. (D) Probe trial data. The percentage of the total time spent in the target quadrant was significantly different between the four groups [*P* = 0.012; *F*(3, 32) = 4.28], and post hoc comparison indicated there was a significant difference between PBS-treated PS2Tg2576 mice and COA-Cl-treated PS2Tg2576 mice. *P < 0.05.

**Fig. 3:**
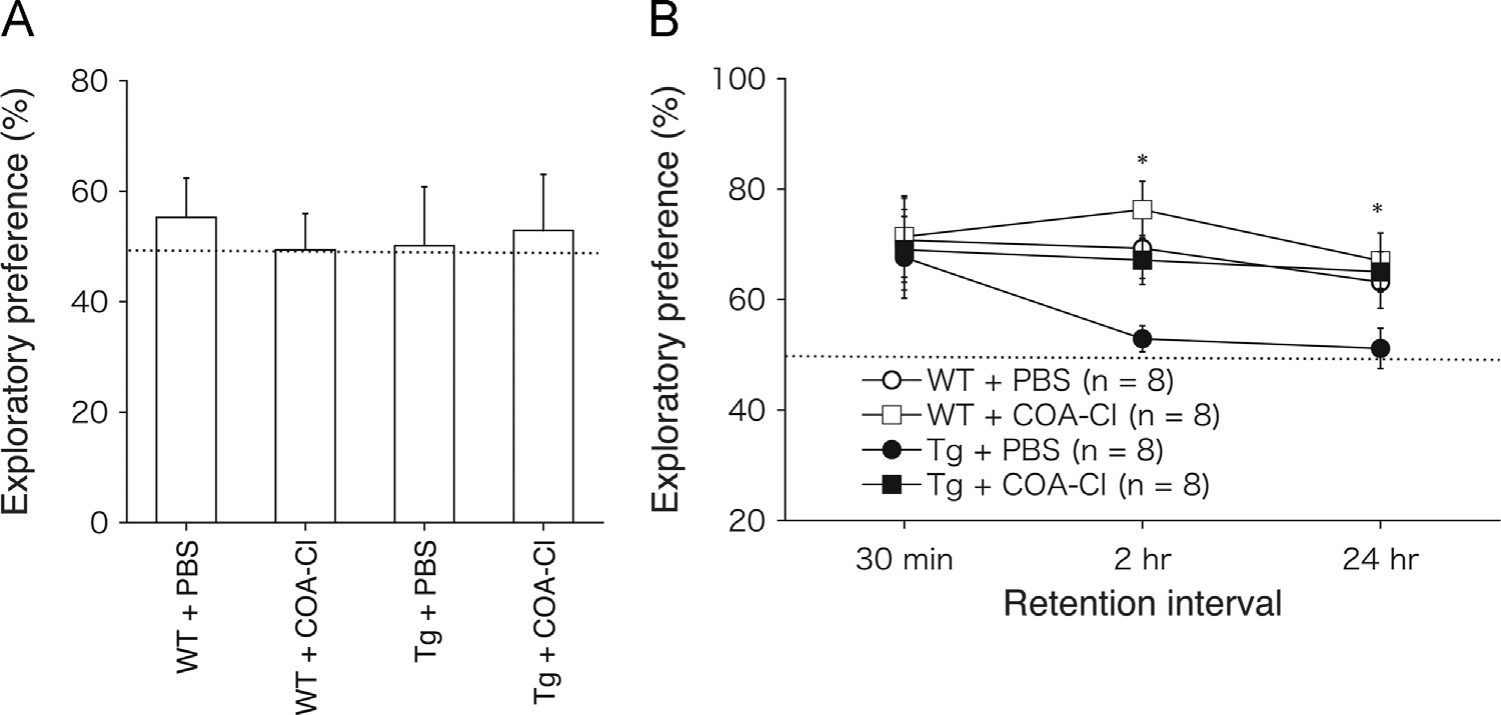
Data showing ameliorated non-spatial-memory deficits in PS2Tg2576 mice after COA-Cl treatment. (A, B) Novel object recognition test in wild-type and PS2Tg2576 mice after COA-Cl treatment. The ordinate represents a ratio of time exploring the displaced novel object to total time spent exploring either of the two objects. The dotted line indicates performance at chance level (50%). (A) Exploratory preference in the training session. (B) The memory of PBS-treated WT mice (open circle, *n* = 8), COA-Cl-treated WT mice (open square, *n* = 8), PBS-treated PS2Tg2576 mice (closed circle, *n* = 8), and COA-Cl-treated PS2Tg2576 mice (closed square, *n* = 8) assessed at three times points after the training session. Recognition memory is expressed in terms of exploratory preference in the retention test. ANOVA revealed no significant interaction effects between sessions and groups [*P* = 0.694; *F*(6, 56) = 0.645], but a significant group effect was observed [*P* = 0.042; *F*(3, 28) = 3.109]. Post hoc comparison indicated a significant difference between PBS-treated PS2Tg2576 mice and COA-Cl-treated PS2Tg2576 mice at 2 h and 24 h after the training session. Significant differences were also observed between PBS-treated WT mice and PBS-treated PS2Tg2576 mice. *P < 0.05.

We sought to determine whether COA-Cl, a novel synthesized nucleoside analog that has been shown to promote angiogenesis and features neuroprotective roles [1], [2], induced behavioral or cognitive changes in PS2Tg2576 mice. First, we collected data regarding spontaneous physical activities in COA-Cl-treated PS2Tg2576 and their control wild-type mice (Fig. 1). Next, data from the Morris water maze (MWM) tests were then collected in the PS2Tg2576 and their control wild-type mice (Fig. 2). Furthermore, we assessed changes in the non-spatial learning ability of the PS2Tg2576 and their control wild-type mice after COA-Cl treatment (Fig. 3).

## 2 Experimental design, materials, and methods

### 2.1 Animals

APP/PS2 double transgenic (PS2Tg2576) mice (*n* = 52) and the littermate wild-type mice (*n* = 52) aged 5–6 months were used. To produce PS2Tg2576 and littermate wild-type mice, male Tg2576 mice in the C57BL6 SJL background (purchased from Taconic Farms, Inc., Hudson, NY USA) were crossed with female PS2 mice, which express human PS2 proteins containing the N141I mutation in the C57BL/6JJcl background (purchased from Immuno-Biological Laboratories Co, Ltd., Fujioka, Japan) [3], [4], [5]. Genotype was confirmed by polymerase chain reaction amplification of genomic DNA extracted from the tail of each mouse, using specific primers for PS2M1 (5′-CGG CTC TAG AGC CTC TGC TAA C-3′ and 5′-CTC TGT GTA GAA GCG CAC AGA C-3′) and Tg2576 (5′-CTG ACC ACT CGA CCA GGT TCT GGG T-3′ and 5′-GTG GAT AAC CCC TCC CCC AGC CTA GAC CA-3′) [5]. PCR was performed under the following 3 sets of conditions: (1) 1 cycle at 94 °C for 2 min; (2) 35 cycles at 98 °C for 10 s, 60 °C for 30 s, and 72 °C for 1 min; and (3) 1 cycle of 72 °C for 5 min [5]. We selected PS2Tg2576 mice as the doubly positive mice [3], [5]. The mice were housed in a room with controlled humidity, temperature, 12/12 h light/dark cycle. Behavioral experiments were performed during the light phase of the light/dark cycle. All animal procedures were approved by the Tokushima Bunri University animal ethics committee and were carried out in accordance with the National Institutes of Health guide for the care and use of laboratory animals.

### 2.2 Drug treatment

PS2Tg2576 mice were divided into two groups: experimental, COA-Cl-treated (*n* = 24) and PBS-treated (*n* = 28). Littermate wild-type mice were also divided into two groups (COA-Cl treated, *n* = 26 and PBS-treated, *n* = 26). The former was intraperitoneally (*i.p*.) injected with 12 mg/kg day of COA-Cl for 3 consecutive days, while the latter was injected *i.p*. with an equal volume of PBS over the same time course.

### 2.3 Spontaneous physical activities in the home cage

**Fig. 4:**
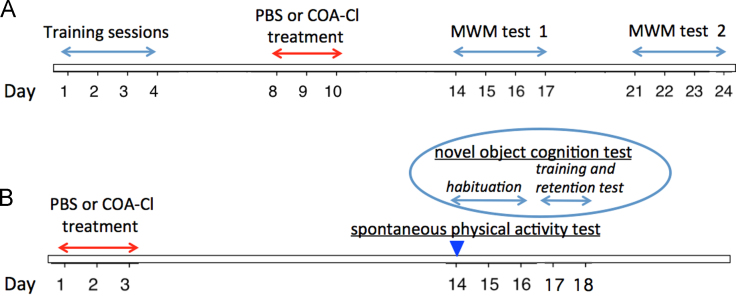
Experimental design for the COA-Cl treatment and behavioral tasks. Timeline of the behavioral test. (A) MWM. The first day of the training phase of MWM was designated as day 1. All mice received behavioral training for four days (from day 1 to day 4). The PS2Tg2576 or wild-type mice were injected intraperitoneally with COA-Cl or PBS for 3 consecutive days (days 8–10). The MWM task was used to assess the behavioral performance of the mice across three sessions following treatment (days 14–17 or 21–24). Probe trial tests were performed one hour after the last behavioral examination on day 17 or day 24. (B) Spontaneous physical activities and novel object recognition tests. Another set of mice was prepared for these tests. The first day of the COA-Cl or PBS treatment was designated as day 1.

Spontaneous physical activity was assessed in the home cage as previously described [6]. Mice were transferred to new home cages (21 × 31 × 12 cm) identical to their original cages, and they were filmed over a period of 3 h, from 09:00 a.m. to 12:00 p.m. The recorded data were analyzed with the HomeCageScan system (CleverSys, Inc., Reston, VA, USA). Spontaneous locomotor activities, such as rearing and distance traveled, were evaluated in one-day period 11 days after the last day of COA-Cl or PBS injection (Fig. 4B).

### 2.4 MWM

Hippocampus-dependent spatial learning ability was evaluated by The MWM task as previously described [7], [8]. Training trials were conducted over 4 days; on each day, four hidden platform tasks were performed with at least 1 h between sequential tasks. A two-minute probe trial test was conducted 1 h after the last trial, prior to which the platform was removed from the pool. COA-Cl or PBS injections were administered 4 days after the training was concluded for 3 consecutive days, as aforementioned. MWM tests were performed starting on either 4 or 11 days following the last day of drug administration (days 14–17 and 21–24, respectively) (Fig. 4A). The behavioral tests took 4 days to conduct: two hidden platform trials per day with at least 1 h between sequential tasks. The probe tests were performed 1 h after the last trial at 7 or 14 days following the last day of injection. We further conducted the visible platform version of the MWM 24 h after the last probe trial. Performance was monitored and analyzed with an automated video-tracking system (Clever System, Inc., Reston, VA).

### 2.5 Novel object recognition test

The experimental protocol was same as described previously with slight modifications [6]. Mice were individually acclimated in an open-field box for 3 days (from 11 days after COA-Cl or PBS injection, Fig. 4B). During training sessions, two novel objects were placed in an open field and the mouse was allowed to explore for 5 min. The time spent exploring each object was recorded. Retention tests were conducted in the same box; however, one of the objects from the training session was replaced with a novel object. The retention assessments were performed 30 min, 2 h, or 24 h after the first interaction, and the mice were again allowed to explore freely for 5 min. A video-tracking system (Clever System, Inc., Reston, VA) was used to obtain the preference index: a ratio of the amount of time spent exploring any one of the two objects (training session) or the novel one (retention session) over the total time spent exploring both objects.

### 2.6 Statistical analysis

The statistical Package for the GraphPad Prism 6 (GraphPad Software Inc., La Jolla, CA, USA) was used to analyze the data for the behavioral tests. Data were expressed as mean ± SEM and analyzed using unpaired two-tailed t-tests or analysis of variance (ANOVA), which was followed by a post hoc Tukey-Kramer or Bonferroni multiple comparison tests. Significance was assigned at *P* < 0.05.
