# Supplementary material for: Data on COA-Cl administration to the APP/PS2 double-transgenic mouse model of Alzheimer׳s disease: Improved hippocampus-dependent learning and unchanged spontaneous physical activity
Source: Data Brief. 2018 Sep 19;20:1877–83. doi: 10.1016/j.dib.2018.09.044 (PMC6168792; doi:10.1016/j.dib.2018.09.044)
Supplement: Supplementary file 1 — Transparency document [file mmc1.doc]

Conflict of Interest and Authorship Conformation Form

Please check the following as appropriate:

- All authors have participated in (a) conception and design, or analysis and interpretation of the data; (b) drafting the article or revising it critically for important intellectual content; and (c) approval of the final version.
- This manuscript has not been submitted to, nor is under review at, another journal or other publishing venue.
- All authors have no affiliation with any organization with a direct or indirect financial interest in the subject matter discussed in the manuscript

Author’s name Affiliation

Yasushi Kishimoto Tokushima Bunri University

Ikuko Tsukamoto Tokushima Bunri University

Atsuko Nishigawa Tokushima Bunri University

Akiko Nishimoto Tokushima Bunri University

Yutaka Kirino Tokushima Bunri University

Yoshihisa Kato Tokushima Bunri University

Ryoji Konishi Kagawa University

Tokumi Maruyama Tokushima Bunri University

Ryoji Konishi Kagawa University

Norikazu Sakakibara Tokushima Bunri University
